# Association of cardiorespiratory fitness with risk of adverse cardiovascular outcomes in cancer patients: a cohort study

**DOI:** 10.3389/fcvm.2025.1569944

**Published:** 2025-05-22

**Authors:** Yutong Wang, Xinyang Song, Tao Xu, Yanwen Chen, Ying Guo, Fang Wang

**Affiliations:** ^1^ Cardiology Department, Beijing Hospital, National Center of Gerontology, Institute of Geriatric Medicine, Chinese Academy of Medical Sciences & Peking Union Medical College, Beijing, China; ^2^ Cardiology Department, Peking University Fifth School of Clinical Medicine, Beijing, China; ^3^ Cardiology Department, Beijing Hospital, National Center of Gerontology, Beijing, China

**Keywords:** physical activity, V̇O_2max_ (maximal oxygen uptake), cardiorespiratory fitness, cancer rehabilitation, physical exercise

## Abstract

**Background:**

Cardiorespiratory fitness (CRF) has gained attention for its potential association with cardiovascular risks. This study aims to examine the association between CRF and adverse cardiovascular outcomes [atrial fibrillation (AF), heart failure (HF), cancer therapy-related cardiovascular toxicity (CTR-CVT)] in cancer patients and explore whether enhancing CRF could improve these outcomes.

**Methods:**

The association between three key exposure factors of CRF—resting heart rate (RHR), maximum heart rate (HRmax), and maximal oxygen uptake (V̇O_2max_)—and the risk of adverse cardiovascular outcomes were assessed using Cox regression analysis.

**Results:**

RHR is significantly associated with an increased risk of adverse events (HF: HR = 1.013, 95% CI 1.008–1.019, *P* < 0.001; CTR_CVT: HR = 1.006, 95% CI 1.004–1.008, *P* < 0.001), except for AF (HR = 0.998, 95% CI 0.994–1.001, *P* = 0.194). HRmax was associated with a lower risk of CTR_CVT events (HR = 0.995, 95% CI 0.993–0.998, *P* < 0.001), but this was not the case for HF and AF (HF: HR = 1.000, 95% CI 0.992–1.009, *P* = 0.962; AF: HR = 0.998, 95% CI 0.992–1.003, *P* = 0.420). V̇O_2max_ was negatively correlated with all adverse events, with HRs ranging from 0.957 to 0.958 (*P* < 0.05 for all). RHR showed a significant non-linear relationship with CTR_CVT (Pnon-linear < 0.0001), AF (Pnon-linear < 0.0001), and HF (Pnon-linear = 0.0057). Similarly, V̇O_2max_ demonstrated a notable non-linear relationship with CTR_CVT (Pnon-linear = 0.0081) and AF (Pnon-linear = 0.0093). No non-linear relationship between HRmax and the outcomes was observed.

**Conclusion:**

Cardiorespiratory fitness, as measured by V̇O_2max_, is consistently negatively correlated with all adverse cardiovascular outcomes, suggesting that higher fitness levels are protective. These findings highlight the potential role of CRF in predicting cardiovascular risks in cancer patients, underscoring the importance of monitoring and improving physical fitness to mitigate adverse outcomes.

## Introduction

Worldwide, cardiovascular disease and cancer are the primary causes of morbidity and mortality (1, 2). Cardiovascular adverse events are frequently observed in oncology patients and represent significant complications that may impact treatment outcomes and overall survival (3, 4). Recent advances in cardiovascular oncology have suggested that shared genetic factors may contribute to the interlinked pathophysiological mechanisms underlying these two conditions (5, 6). Cancer treatments may induce cardiovascular toxicity, which in turn may aggravate shared risk factors for both cardiovascular disease and cancer (7). Meanwhile, not only cardiovascular dysfunction itself may promote tumor progression, cardiovascular-related complications are also a significant cause of non-cancer mortality in cancer patients (8). Moreover, advancements in medical care have led to increased survival rates among cancer years, which has consequently resulted in a rise of secondary cardiovascular diseases. In some cases, the success of cancer treatment is achieved at the cost of compromised cardiac function (9).

Oncology patients frequently experience a range of cardiovascular adverse events. Atrial fibrillation (AF), the most common arrhythmic disorder, appears to share underlying pathophysiological mechanisms and risk factors with cancer (10). Heart failure (HF) is another significant cardiovascular complication in cancer patients. Anti-cancer treatments can increase the risk of HF (11, 12), while biomarkers of HF can serve as prognostic predictors for cancer patients (13). In addition to these common cardiac complications, several other adverse cardiac outcomes, such as arrhythmias, hypertension, and vascular toxicity, have been identified and collectively termed cancer therapy-related cardiovascular toxicity (CTR-CVT) in the 2022 guidelines on cardio-oncology (14), which encompasses the majority of adverse cardiovascular outcomes in cancer patients. These cardiac toxicities not only affect the prognosis of cancer patients but also should be carefully considered by medical professionals during cancer treatment and rehabilitation.

Nowadays, with socioeconomic development and growing health awareness, proactive health strategies beyond conventional treatments such as medications and surgeries are gaining increasing attention. Among these, regular and scientifically guided physical exercise has emerged as a key area of focus. Both European Society of Cardiology (ESC) and American Heart Association (AHA) guidelines recommend regular moderate exercise for adults; however, the benefits and potential risks of physical activity vary among individuals, particularly those with underlying conditions (15).

Individuals can adjust the amount and intensity of exercise based on their physical condition, which limits the use of exercise intensity or duration alone to assess long-term benefits. In contrast, V̇O_2max_, as a relatively stable indicator of physical fitness (though it can be improved with a structured exercise program), reliably reflects its relationship with prognosis and the improvements in outcomes following its enhancement. The direct measure of cardiorespiratory fitness is maximal oxygen uptake (V̇O_2max_), typically assessed via cardiopulmonary exercise testing (CPET) (16). However, due to the inconvenience and difficulty of CPET, it is not a routine clinical examination, and V̇O_2max_ is often estimated using formulas (17, 18). As a comprehensive indicator of overall metabolism and cardiovascular health, CRF has been shown to be closely associated with cardiovascular outcomes in the general population (19–21). However, large-scale, high-quality prospective studies exploring the potential of CRF as a predictor of adverse cardiovascular outcomes in cancer patients are currently lacking. Additionally, existing research has primarily focused on the mechanisms and interventions related to treatment-induced cardiac toxicity (e.g., anthracyclines, targeted therapies), with limited investigation into baseline CRF in cancer patients. While some studies indicate that cancer patients generally have reduced exercise tolerance, potentially due to treatment side effects and associated inflammation, the impact on long-term cardiovascular outcomes remains inadequately assessed (22, 23). This study focuses on the intersection of cancer and cardiovascular health, specifically investigating the predictive value of CRF for adverse cardiovascular outcomes in cancer patients. By highlighting the significance of integrating expertise from oncology and cardiology, the study underscores the necessity of adopting a multidisciplinary approach to optimize patient management. Furthermore, it provides evidence supporting personalized cardiovascular risk assessment and intervention strategies. For patients, the findings advocate for improving CRF as a means to enhance quality of life and reduce the risk of adverse cardiovascular events.

## Methods

### Data source and study participants

This study utilized data from the UK Biobank, a large biomedical resource that includes comprehensive genetic, clinical, and health information from approximately 500,000 participants aged 37–73 years. Baseline data were collected between 2006 and 2010 across 22 assessment centers throughout the UK, with continuous follow-up evaluations. The UK Biobank was approved by the North West Multi-Centre Research Ethics Committee (approval number 11/NW/0382), and all participants provided written informed consent. We included all patients diagnosed with cancer under the ICD-10 codes at baseline, encompassing all types of cancer, whether a single cancer or multiple distinct cancers. We excluded individuals who: (1) withdrew consent from the UK Biobank, (2) lacked cardiorespiratory fitness data, (3) had missing covariate data, or (4) had a history of atrial fibrillation, heart failure, or CTR_CVT, or had died at baseline. Ultimately, 4,949 participants were included in the final analysis (Supplementary Figure 1).

### Assessment of exposure

The submaximal cycling ergometer test was conducted using a stationary electric bicycle equipped with firmware version 1.7. Participants were assigned personalized exercise protocols, and electrocardiograms (ECG) were recorded at four distinct stages using the CAM-USB 6.5 device with Cardiosoft software version 6.51: a pre-test stage (15 s), a constant stage (2 min, with 30 W for females and 40 W for males), an incremental stage (4 min, with workloads increasing to 35% of the estimated maximum workload for low-risk participants and 50% for higher-risk participants), and a recovery stage (1 min).

The maximum workload was estimated using a predictive model incorporating age and physical examination findings. The maximum heart rate represents the maximum heart rate recorded in the activity phase, during which the participant cycles for a specified duration with increasing cycling intensity, in bike exercise test. Notably, the UK Biobank limits the maximum ergometer value to 50% of the expected maximum workload. Furthermore, the linear relationship between heart rate and workload may not hold at maximum heart rate, particularly in older individuals (24). The predicted MaxHR was calculated using an established formula in prior publication (25). Resting heart rate was measured via ECG while participants were at rest. These data were then used to estimate V̇O_2max_ (17, 18).

### Assessment of outcomes

Atrial fibrillation, heart failure, and CTR_CVT outcomes were identified using three-digit ICD-10 codes, a validated method for disease classification and distinction. The conditions included in CTR_CVT were defined according to the recent guideline (14). Follow-up began on the recruitment date and continued until the earliest occurrence of the first disease diagnosis, death, loss to follow-up, or the exclusion date.

### Covariates

This study assessed several covariates, including physical measurements, sociodemographic characteristics, lifestyle factors, and medical history. Sociodemographic factors included gender (male or female), age, and ethnicity (white or non-white). Lifestyle factors comprised smoking status (current, former, or never smoked), alcohol consumption (current, former, or never drank), and dietary quality. Dietary quality was evaluated using a previously reported diet score, categorized as healthy, moderate, or unhealthy based on intake of fruits, vegetables, red or processed meats, and salt (26). Physical measurements included systolic and diastolic blood pressure (SBP and DBP, measured using an electronic sphygmomanometer and the average of two measurements was recorded), waist-to-hip ratio (WHR, waist and hip circumferences were measured physically and the waist-to-hip ratio was then calculated), and body mass index (BMI, measured at baseline using bioelectrical impedance). Medical history included a history of cardiovascular disease in parents, as well as personal use of antihypertensive, lipid-lowering, and antidiabetic medications.

### Statistical analysis

Baseline demographic and medical characteristics were stratified by gender. Continuous variables are presented as means with standard deviations, while categorical variables are reported as frequencies and percentages.

Longitudinal risk analysis was performed using Cox proportional hazards models to assess the associations between resting heart rate, maximum heart rate, and V̇O_2max_ with the risk of adverse outcomes. Hazard ratios (HR) and 95% confidence intervals (CI) were reported. Dose-response relationships between these factors and adverse outcomes were evaluated using three-knot restricted cubic spline (RCS) models. All analyses were adjusted for the covariates.

Statistical significance was defined as a two-sided *P* value less than 0.05. Data analysis and visualization were conducted using R software (version 4.2.1).

## Results

Table 1 presents the baseline characteristics of the study population, grouped by gender. Males had higher WHR, BMI, and blood pressure compared to females, as well as higher smoking and alcohol consumption rates. Additionally, a greater proportion of males had a family history of CVD, which could contribute to gender differences in adverse cardiovascular outcomes. Notably, however, there were no significant differences in cardiorespiratory fitness between males and females.

**Table 1:** Baseline characteristics.

Characteristics	Male	Female
Sample size, *n*	1,647	3,302
Age, years	61.09 (6.73)	58.62 (7.54)
Ethnic, white, *n* (%)	1,573 (95.97)	3,122 (94.81)
College, *n* (%)	600 (36.81)	1,059 (32.45)
WHR	0.94 (0.06)	0.82 (0.07)
BMI, kg/m^2^	27.47 (3.99)	26.54 (4.83)
SBP, mmHg	141.61 (17.35)	135.95 (19.05)
DBP, mmHg	83.12 (9.28)	80.31 (9.83)
TDI, *n* (%)		
Low	557 (33.82)	1,019 (30.91)
Intermediate	571 (34.67)	1,156 (35.06)
High	519 (31.51)	1,122 (34.03)
Diet quality, *n* (%)		
Healthy	49 (3.00)	269 (8.20)
Intermediate	1,216 (74.37)	2,618 (79.82)
Unhealthy	370 (22.63)	393 (11.98)
Smoking status, *n* (%)		
Never	784 (47.86)	1,870 (57.03)
Previous	694 (42.37)	1,142 (34.83)
Current	160 (9.77)	267 (8.14)
Alcohol consumption, *n* (%)		
Never	50 (3.04)	173 (5.25)
Previous	45 (2.74)	110 (3.34)
Current	1,550 (94.22)	3,012 (91.41)
History of CVD, *n* (%)	632 (38.37)	1,032 (31.25)
RHR, beats per minute	62.08 (10.60)	62.87 (9.48)
HRmax, beats per minute	107.45 (17.50)	112.60 (20.19)
Estimated V̇O_2max_, ml (kg·min)	18.24 (4.56)	18.24 (4.56)

Continuity and categorical variables are shown as mean (SD) and number (percentage), respectively.

WHR, waist-to-hip ratio; BMI, body mass index; SBP, systolic bloodpressure; DBP, diastolic blood pressure; TDI, Townsend deprivation index; CVD, cardiovascular disease; RHR, resting heart rate; HR, heart rate; $\dot{V}O_{2\max}$, maximal oxygen uptake.

Table 2 illustrates the relationship between cardiopulmonary fitness, heart rate, and cardiovascular adverse events in cancer patients. RHR was positively correlated with adverse outcomes, with a higher RHR significantly associated with an increased risk of adverse events (HF: HR = 1.013, 95% CI 1.008–1.019, *P* < 0.001; CTR_CVT: HR = 1.006, 95% CI 1.004–1.008, *P* < 0.001), except for AF (HR = 0.998, 95% CI 0.994–1.001, *P* = 0.194). Conversely, HRmax was negatively correlated with CTR_CVT, where a higher HRmax was associated with a lower risk of CTR_CVT events (HR = 0.995, 95% CI 0.993–0.998, *P* < 0.001), but this was not the case for HF and AF (HF: HR = 1.000, 95% CI 0.992–1.009, *P* = 0.962; AF: HR = 0.998, 95% CI 0.992–1.003, *P* = 0.420). Furthermore, V̇O_2max_ was negatively correlated with the occurrence of all adverse events. Better V̇O_2max_ was associated with a reduced risk of adverse outcomes, with HRs ranging from 0.957 to 0.958 (*P* < 0.05 for all).

**Table 2:** Hazard ratios for all the outcomes.

Outcomes	Resting heart rate		Maximum heart rate		$\dot{V}O_{2\max}$	
	HR (95% CI)	*P* value	HR (95% CI)	*P* value	HR (95% CI)	*P* value
HF	1.013 (1.008–1.019)	<0.001	1.000 (0.992–1.009)	0.962	0.957 (0.921–0.995)	0.026
AF	0.998 (0.994–1.001)	0.194	0.998 (0.992–1.003)	0.420	0.958 (0.934–0.982)	<0.001
CTR_CVT	1.006 (1.004–1.008)	<0.001	0.995 (0.993–0.998)	<0.001	0.957 (0.946–0.969)	<0.001

The Cox proportional hazard models were adjusted for age, sex, ethnicity, education, Townsend deprivation index, smoking status, alcohol consumption, diet quality, blood pressure, waist-to-hip ratio, body mass index, parental history of cardiovascular disease, personal history of antihypertensive, lipid-lowering, and glucose-lowering medication use.

AF, atrial fibrillation; HF, heart failure; $\left(\dot{V}O_{2\max}\right)$: maximal oxygen consumption; CTR-CVT: cancer therapy-related CV toxicity.

Notably, Supplementary Table 1 shows the associations between the maxHR/predicted maxHR ratio and these three outcomes. In this cohort, 1.5% of participants achieved ≥85% of their age-predicted maxHR during exercise testing. A higher ratio showed a significant inverse association with the risk of CTR-CVT (HR = 0.466, 95% CI: 0.311–0.698, *P* < 0.001). In contrast, no statistically significant associations were observed between the ratio and the incidence of AF or HF.

**Figure 1:**
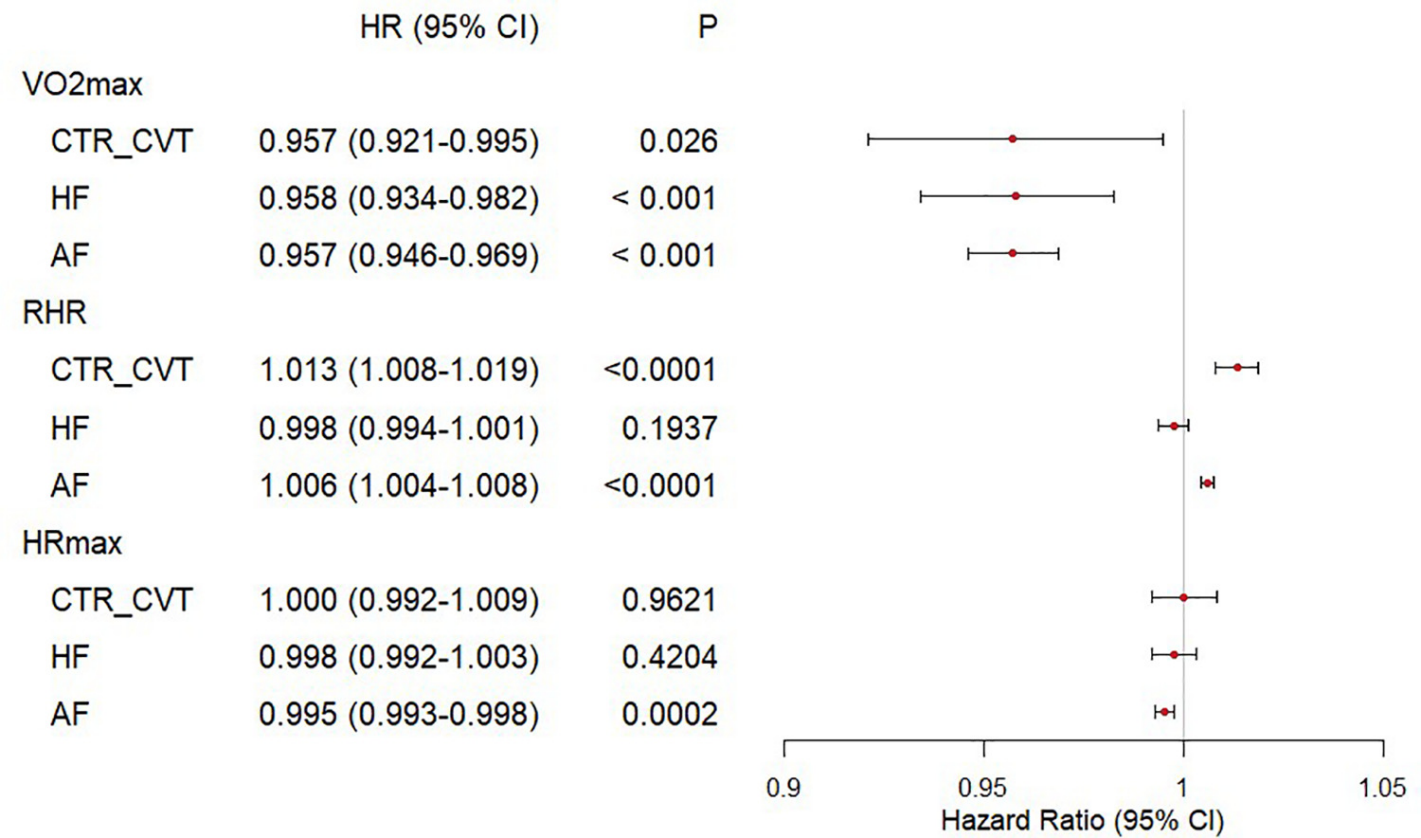
Forest plot of cardiorespiratory fitness and risk of incident HF, AF, CTR_CVT in participants with cancer. The Cox regression was used to draw the forest plot of cardiorespiratory fitness and outcomes. The HR and their 95% CI were reported. The Cox proportional hazard models were adjusted for The Cox proportional hazard models were adjusted for age, sex, ethnicity, education, Townsend deprivation index, smoking status, alcohol consumption, diet quality, blood pressure, waist-to-hip ratio, body mass index, parental history of cardiovascular disease, personal history of antihypertensive, lipid-lowering, and glucose-lowering medication use.

Figure 1 presents a forest plot that visually summarizes the results above. Higher V̇O_2max_ values were associated with a significantly lower hazard ratio for adverse outcomes, which suggests a consistent protective effect of V̇O_2max_. Elevated RHR showed an increased risk in the CTR_CVT and AF groups, while no significant association was observed in the HF group. HRmax shows minimal impact except for a small effect in AF.

**Figure 2:**
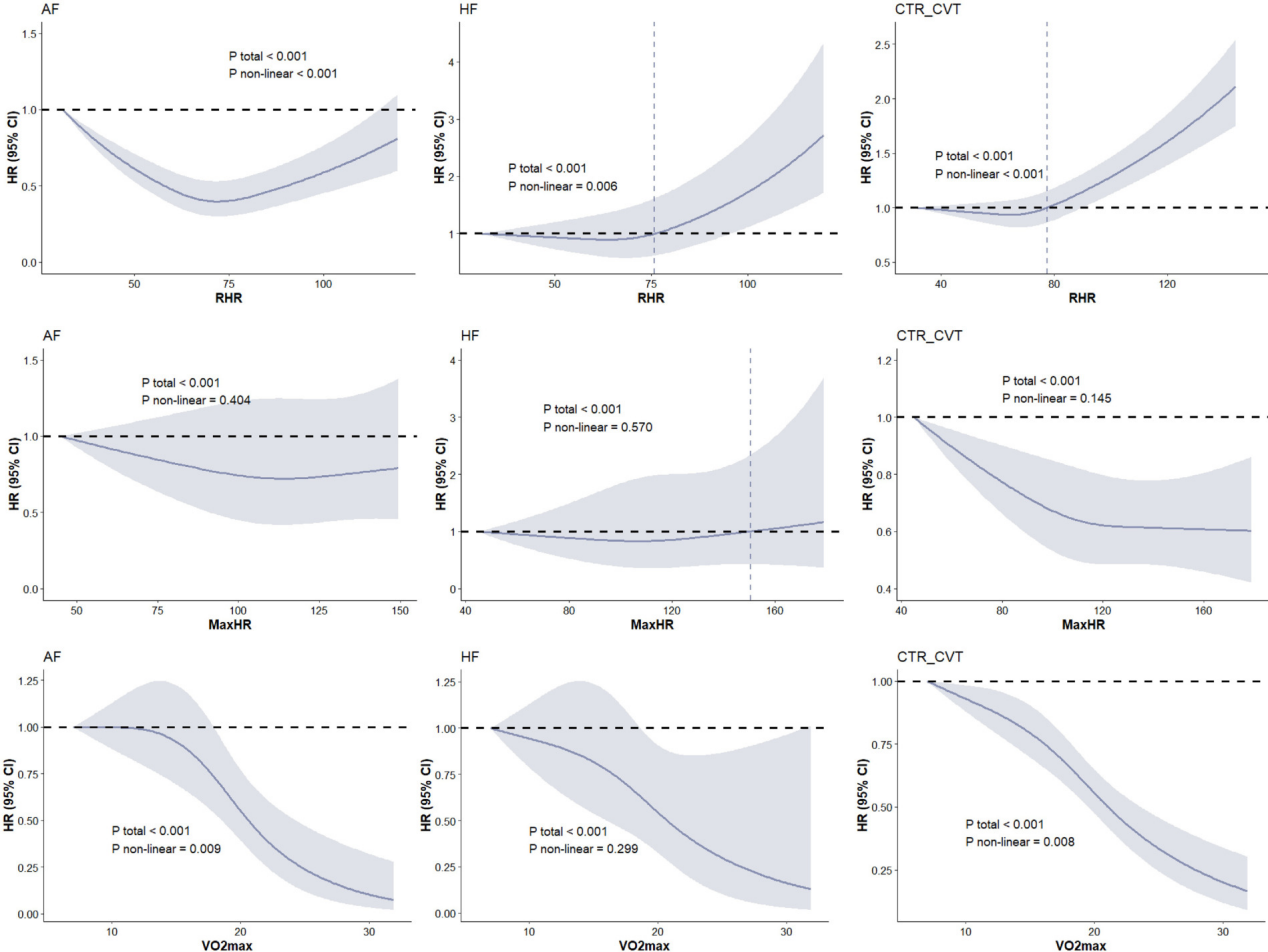
Dose-response associations between cardiorespiratory fitness and risk of incident HF, AF, CTR_CVT in participants with cancer. The Cox regression models with restricted cubic splines were used to explore the dose-response relationship between cardiorespiratory fitness and outcomes. The HR and their 95% CI were reported. The Cox proportional hazard models were adjusted for The Cox proportional hazard models were adjusted for age, sex, ethnicity, education, Townsend deprivation index, smoking status, alcohol consumption, diet quality, blood pressure, waist-to-hip ratio, body mass index, parental history of cardiovascular disease, personal history of antihypertensive, lipid-lowering, and glucose-lowering medication use.

Figure 2 displays the restricted cubic spline (RCS) curves illustrating the relationship between cardiopulmonary fitness, heart rate, and the probability of cardiovascular adverse events. RHR showed a significant non-linear relationship with CTR_CVT (Pnon-linear < 0.0001), AF (Pnon-linear < 0.0001), and HF (Pnon-linear = 0.0057). Similarly, V̇O_2max_ demonstrated a notable non-linear relationship with CTR_CVT (Pnon-linear = 0.0081) and AF (Pnon-linear = 0.0093). However, no non-linear relationship between HRmax and the outcomes was observed.

## Discussion

The main findings of this study are as follows. First, higher RHR was associated with an increased risk of HF and CTR-CVT, while higher HRmax was linked to a lower risk of CTR-CVT. Specifically, cancer patients with an RHR of 73 had the lowest risk of HF, those with an RHR of 65 had the lowest risk of CTR-CVT, and those with an RHR of 72 had the lowest risk of AF. Finally, higher V̇O_2max_ levels were significantly associated with reduced risks of AF, HF, and CTR-CVT, highlighting its important predictive value for cardiovascular outcomes. Our findings suggest that, overall, cardiorespiratory fitness, a concept commonly discussed in sports and exercise, plays a critical role in cardiovascular and rehabilitation medicine. Resting heart rate and maximum heart rate during exercise can serve as indicators of cardiorespiratory fitness, thereby helping predict adverse cardiovascular outcomes. Notably, V̇O_2max_ was found to be associated with an increased risk of cardiovascular adverse events, such as AF and HF, in cancer patients, independent of major confounders like family history and lifestyle factors. It suggests that cancer patients with better cardiorespiratory fitness—indicated by a lower RHR, higher HRmax during exercise, and higher V̇O_2max_—may derive the greatest benefits in reducing cardiovascular adverse outcomes and maintaining cardiovascular health. In contrast, patients with poorer cardiorespiratory fitness are more likely to experience adverse cardiovascular outcomes.

CRF is not only a well-established concept in the field of sports and exercise but is also gaining significant recognition in medicine and rehabilitation. It serves as a comprehensive indicator of cardiovascular health, reflecting overall cardiopulmonary efficiency, autonomic regulation, and the dynamic functioning of the heart (27). Cardiac function is crucial in determining the prognosis and outcomes of cancer patients, particularly in advanced stages, where cardiac burden and cardiotoxicity are often exacerbated (28). Therefore, an intuitive method is needed to assess the association between cardiac function and cardiovascular events in cancer patients. Clinically, CRF provides a comprehensive evaluation of patient's cardiovascular health and informs the development of targeted therapeutic and intervention strategies.

RHR refers to the number of heart beats per minute measured while at complete rest and relaxation, typically recommended to be taken upon waking. It serves as an indicator of heart health and cardiovascular function. An elevated RHR increases myocardial oxygen consumption, enhances sympathetic nervous activity, and may exacerbate endothelial dysfunction (29, 30). These changes can lead to intensified vasoconstriction, accelerated atherosclerosis, elevated blood pressure, and myocardial hypertrophy, ultimately contributing to adverse cardiovascular outcomes (31). However, studies examining the relationship between RHR and the incidence of cardiovascular events and mortality remain contentious, with no definitive mechanisms established (32). Some studies have shown that cancer patients typically have a higher RHR compared to healthy individuals, and an elevated RHR is a strong and independent predictor of mortality (33–35). This finding may support the use of personalized treatment strategies, such as medications aimed at maintaining heart rate in patients.

HRmax is the highest heart rate an individual can achieve during intense or vigorous exercise and is an important indicator of cardiac reserve. A lower HRmax may suggest limited cardiac reserve, restricting the ability to increase cardiac output and increasing hemodynamic strain, which can indicate a higher risk of cardiovascular diseases, such as heart failure. Therefore, assessing HRmax can be crucial for cardiovascular risk stratification and the development of rehabilitation plans, particularly as there is growing emphasis on healthy lifestyle strategies. Notably, only 1.5% of participants in this cohort achieved ≥85% of their age-predicted maxHR during exercise testing. Failure to reach the target heart rate may indicate impaired cardiac function and reduced cardiovascular reserve. Failure to achieve 85% of the age-predicted maxHR, defined as chronotropic incompetence, has been previously identified as an independent predictor of mortality in coronary artery disease (36, 37). Our study further suggests that assessing whether patients attain their predicted maxHR can provide additional diagnostic value in evaluating cardiopulmonary function and serve as a predictor of cardiovascular adverse events in cancer patients.

V̇O_2max_ is considered the “gold standard” for cardiopulmonary function, reflecting the combined efficiency of cardiac output, oxygen-carrying capacity of the blood, and muscle oxygen utilization (38). Higher V̇O_2max_ is associated with improved insulin sensitivity, reduced inflammation, and enhanced lipid metabolism, all of which contribute to lower cardiovascular risk (39–41). Additionally, research on the long-term prognosis of V̇O_2max_ suggests that it has the potential to become a strong independent predictor of cardiovascular health and all-cause mortality, positioning it as a predictor as traditional risk factors such as blood pressure and cholesterol levels (42). Therefore, improving V̇O_2max_ through aerobic exercise is an important goal in preventing and rehabilitating cardiovascular disease.

Studie proved the role of CRF in cardiovascular diseases, and showed association between CRF and cancer incidence (43, 44). This study further confirms the effectiveness of CRF in evaluating cardiovascular prognosis in cancer patients, complementing existing research and thereby broadening the scope of integrative medicine and interdisciplinary applications. Evidence now supports physical activity and exercise as primary prevention strategies for cancer patients (45). Regular, moderate exercise not only directly enhances cardiopulmonary function, thereby improving cardiovascular health, but also improves body composition and increases muscle mass, which further benefits cardiovascular function (46, 47).

This study focuses on three primary endpoints: atrial fibrillation, hypertension, and CTR_CVT. Notably, AF and HF present a unique clinical challenge. First, managing both AF and cancer patients involves uncertainty regarding bleeding and thrombotic risks, the onset of new AF may serve as a potential risk predictor for cancer (48, 49). Secondly, cancer and HF share common risk factors (49–51), possible mechanisms include inflammation and oxidative stress, neuro-hormonal and cytokine activation, and immune system dysfunction (52). The risk of cancer in HF patients increases over time (53–56), with the underlying cancer condition further exacerbating the mortality risk in those affected by HF (57–60). Therefore, further research is needed to investigate the relationship between them.

While it covers most cardiovascular conditions commonly encountered by cancer patients during treatment and recovery, it does not encompass all known outcomes. Due to data limitations, more detailed disease classifications could not be obtained, such as identifying specific immune-mediated myocardial inflammation caused by different immunosuppressants. Additionally, the study lacks detailed ECG diagnoses, preventing the identification of patients with prolonged QT intervals. Also, bradycardia and Raynaud's syndrome were not included in the analysis. Moreover, the study does not specify the exact treatments, chemotherapy regimens, or medications received by the patients. Therefore, further targeted research is needed.

Our data suggest that implementing appropriate interventions for cancer patients with low cardiorespiratory fitness can significantly enhance individual capabilities, improving both clinical effectiveness and cost efficiency. Therefore, healthcare providers and community workers can promote moderate aerobic exercise training, high-intensity interval training (HIIT), and other scientifically guided exercise which can improve the cardiorespiratory fitness within the community and among patient populations. This approach not only has the potential to improve patient prognosis but also to enhance their overall quality of life. While cardiorespiratory fitness testing can be challenging to measure in healthcare and community settings, it can be easily estimated using simple methods, making it a practical screening tool for various environments.

## Limitation

Firstly, instead of measuring V̇O_2max_ directly, the data in this study was calculated by a formula. Therefore, there may be some inaccuracies. Nevertheless, the data used for calculations such as heart rate, age or weight were accurately measured, which increased the credibility and reliability of these results. Secondly, there are some issues with the population distribution in this study. The proportion of middle-aged and older adults is slightly higher, which may lead to biased distributions of data such as heart rate, potentially affecting the results. Additionally, the majority of the sample is white people, highlighting the need for future research targeting other ethnic groups. Furthermore, the overrepresentation of females may also introduce bias. While it covers most cardiovascular conditions commonly encountered by cancer patients during treatment and recovery, it does not encompass all known outcomes. Due to data limitations, more detailed disease classifications could not be obtained, such as identifying specific immune-mediated myocardial inflammation caused by different immunosuppressants. Also, the study lacks detailed ECG diagnoses, preventing the identification of patients with prolonged QT intervals. Bradycardia and Raynaud's syndrome were not included in the analysis as well. Additionally, due to limitations in the data format and the primary focus of this study on examining the impact of CRF on adverse cardiovascular outcomes in cancer patients (regardless of the treatment received), this study did not categorize patients based on their specific treatments, chemotherapy regimens, or medications. Anti-tumor drugs may affect the results, while drugs such as beta blockers, which are commonly used, may affect heart rate. Therefore, future research will include more targeted investigations, such as studies focusing on specific types of cancer or cancer patients receiving particular drug treatments.

## Data Availability

The datasets presented in this study can be found in online repositories. The names of the repository/repositories and accession number(s) can be found below: All data in this present study is available in the UK Biobank (https://www.ukbiobank.ac.uk). All data can be viewed online (https://biobank.ndph.ox.ac.uk/showcase/index.cgi), while the use of the data requires an application. The present study was approved by the UK Biobank under application number 144894 (https://ams.ukbiobank.ac.uk/ams/resMessages).
