# Supplementary figures and images for: Association of cardiorespiratory fitness with risk of adverse cardiovascular outcomes in cancer patients: a cohort study
Source: Front Cardiovasc Med. 2025 May 22;12:1569944. doi: 10.3389/fcvm.2025.1569944 (PMC12137253; doi:10.3389/fcvm.2025.1569944)

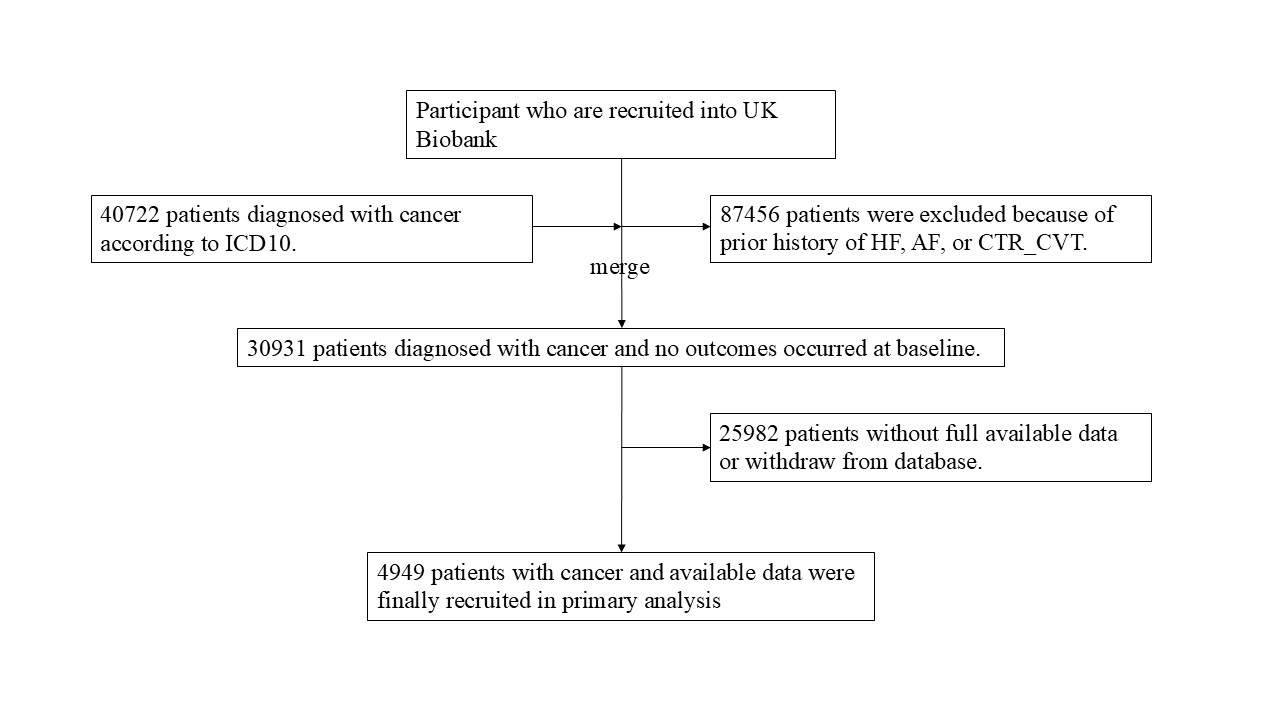

Supplement: Supplementary file 1 [file Image1.jpeg]
