# Supplementary material for: Association of cardiorespiratory fitness with risk of adverse cardiovascular outcomes in cancer patients: a cohort study
Source: Front Cardiovasc Med. 2025 May 22;12:1569944. doi: 10.3389/fcvm.2025.1569944 (PMC12137253; doi:10.3389/fcvm.2025.1569944)
Supplement: Supplementary file 2 [file Table1.docx]

| **Supplementary Table 1. Hazard Ratios for MaxHR/Predicted MaxHR Ratio and All the Outcomes** | | |
| --- | --- | --- |
| outcomes | MaxHR/Predicted MaxHR Ratio | |
|  | *HR (95%CI)* | *P value* |
| HF | 1.043 (0.268-4.063) | 0.9521 |
| AF | 0.700 (0.286-1.714) | 0.4348 |
| CTR_CVT | 0.466 (0.311-0.698) | 0.0002 |
| The Cox proportional hazard models were adjusted for age, sex, ethnicity, education, Townsend deprivation index, smoking status, alcohol consumption, diet quality, blood pressure, waist-to-hip ratio, body mass index, parental history of cardiovascular disease, personal history of antihypertensive, lipid-lowering, and glucose-lowering medication use | | |
| Abbreviation: AF, atrial fibrillation; HF, heart failure; VO2max: maximal oxygen consumption; CTR-CVT: cancer therapy-related CV toxicity | | |
